# Offenders and non-offenders with schizophrenia spectrum disorders: the crime-preventive potential of sufficient embedment in the mental healthcare and support system

**DOI:** 10.3389/fpsyt.2023.1231851

**Published:** 2023-08-30

**Authors:** Lena Machetanz, Andreas B. Hofmann, Jan Möhrke, Johannes Kirchebner

**Affiliations:** ^1^Department of Forensic Psychiatry, University Hospital of Psychiatry Zurich, Zurich, Switzerland; ^2^Department of Psychiatry, Psychotherapy, and Psychosomatics, University Hospital of Psychiatry Zurich, Zurich, Switzerland

**Keywords:** schizophrenia spectrum disorders, offender patients, forensic psychiatry, PANSS, symptom severity, offending

## Abstract

**Background:**

Suffering from schizophrenia spectrum disorder (SSD) has been well-established as a risk factor for offending. However, the majority of patients with an SSD do not show aggressive or criminal behavior. Yet, there is little research on clinical key features distinguishing offender from non-offender patients. Previous results point to poorer impulse control, higher levels of excitement, tension, and hostility, and worse overall cognitive functioning in offender populations. This study aimed to detect the most indicative distinguishing clinical features between forensic and general psychiatric patients with SSD based on the course of illness and the referenced hospitalization in order to facilitate a better understanding of the relationship between violent and non-violent offenses and SSD.

**Methods:**

Our study population consisted of forensic psychiatric patients (FPPs) with a diagnosis of F2x (ICD-10) or 295.x (ICD-9) and a control group of general psychiatric patients (GPPs) with the same diagnosis, totaling 740 patients. Patients were evaluated regarding their medical (and, if applicable, criminal) history and the referenced psychiatric hospitalization. Supervised machine learning (ML) was used to exploratively evaluate predictor variables and their interplay and rank them in accordance with their discriminative power.

**Results:**

Out of 194 possible predictor variables, the following 6 turned out to have the highest influence on the model: olanzapine equivalent at discharge from the referenced hospitalization, a history of antipsychotic prescription, a history of antidepressant, benzodiazepine or mood stabilizer prescription, medication compliance, outpatient treatment(s) in the past, and the necessity of compulsory measures. Out of the seven algorithms applied, gradient boosting emerged as the most suitable, with an AUC of 0.86 and a balanced accuracy of 77.5%.

**Discussion:**

Our study aimed to identify the most influential illness-related predictors, distinguishing between FPP and GPP with SSD, thus shedding light on key differences between the two groups. To our knowledge, this is the first study to compare a homogenous sample of FPP and GPP with SSD regarding their symptom severity and course of illness using highly sophisticated statistical approaches with the possibility of evaluating the interplay of all factors at play.

## 1. Introduction

Unlawful behavior imposes huge social and economic costs on society, affecting individuals, businesses, and institutions. This is especially relevant in regard to violent crimes: interpersonal violence is considered to be among the top 20 leading causes of disability-adjusted life years worldwide, thus posing a highly relevant burden on public health (1, 2). Since 1996, the World Health Assembly has repeatedly emphasized the problematic contribution of fatal and non-fatal interpersonal violence to global mortality and morbidity (3, 4). Apart from the incalculable human toll it takes, violence is also an economic burden. It has been estimated that interpersonal violence accounts for over seven times the cost of collective violence, e.g., in the context of armed conflicts or terrorist attacks (5). However, while the costs of violent crime may seem more tangible, non-violent crimes, such as theft or criminal damage, are by no means trivial offenses without victims (6). As with all criminal acts, they result in direct burdens on the victim, in the form of property damage and loss of value, on the criminal justice system, in the form of correctional programs, and on the perpetrator in the form of lost life opportunities (7). Therefore, with the intent of prevention, research in the past decades has increasingly focused on identifying risk factors for violent and non-violent criminal behaviors. Apart from certain biographical experiences, such as bullying or parental neglect, as well as poor moral judgment, neuropsychiatric disorders have been known to significantly increase both the relative and absolute risks for the perpetration of violent and non-violent criminal behaviors (8–10). Among those, substance use disorders, personality disorders, and psychotic disorders have been shown to have the strongest associations with violent and antisocial behaviors (10–12). While the latter are well-established as risk factors, the majority of all patients suffering from schizophrenia spectrum disorders (SSDs) do not engage in criminal behavior (13, 14). For instance, in a large retrospective study by Seena Fazel and Martin Grann (15), ~33% of all patients with schizophrenia had committed some form of violent offense, thus resulting in an attributable risk fraction of 2.3% (15). It should also be noted that patients suffering from severe mental disorders, in general, and psychotic disorders, in particular, are also at higher risk of victimization, although current research features some methodological difficulties (16, 17). As the occurrence of violence in patients with SSD and the subsequent stigmatization may also contribute to an elevated burden of disease, it is important to identify and address risk factors for violence in those patients (18, 19). In a meta-analysis by Yee et al. (14), only 22% of individuals with psychosis engaged in any form of criminal offending (including non-violent offending) (14), which raises the question of which factors contribute to an individual committing a crime in the course of their SSD, and which factors may have a protective effect. Yet, there is a scarcity of corresponding research: first, previous results often lack consistency regarding the roles of certain risk factors (20). Second, while individuals diagnosed with SSD pose a non-negligible subgroup of offenders, studies have seldom focused on this specific population, evaluating rather diagnostically heterogeneous samples, e.g., psychotic disorders in general (21–24). Those who did often times had a limited number of cases, thus having an increased risk of being subjected to a type II error (25–28). To date, poorer impulse control; higher levels of excitement, tension, and hostility; and worse overall cognitive functioning appear to draw a dividing line between offenders and non-offenders with SSD (20, 25, 26). This, however, leads to another issue: all of these factors are complex, multifactorial, and oftentimes interdependent and have yet to be comprehensively understood. Null hypothesis significance testing or simple linear regressions, assuming linear relationships between variables, may not accommodate the exploration of the complex phenomenon of offending in mental illness.

Therefore, the objectives of this study were as follows:

I) To exploratively evaluate clinical key factors from the patient's history as well as their referenced hospitalization, distinguishing offenders and non-offenders with SSD based on items regarding the underlying mental illness, using supervised machine learning (ML).
II) To quantify the performance of the calculated ML model.

## 2. Materials and methods

The study was approved by the ethics committee in Zurich, Switzerland, under the reference number KEK-ZH-NR 2014–0480 as part of a larger, ongoing project investigating the characteristics of offender patients with SSD.

### 2.1. Participants

Our study group consisted of 370 male and female forensic psychiatric patients (FPPs) with a diagnosis of SSD according to ICD-10 (F2x) or ICD-9 (295.x) (29, 30). All of them had been in court-mandated inpatient treatment at the Center for Inpatient Forensic Therapies of the University Hospital of Psychiatry Zurich, Switzerland, between 1982 and 2016, with 296 of those cases being treated after 2000. The legal basis for such inpatient forensic therapy in Switzerland is provided by Article 59 of the Swiss Penal Code, and patients subjected to court-mandated treatment can either be referred to forensic psychiatric institutions (as in this case) or even to suitable residential facilities and specialized prison departments (31). Offenses leading to forensic psychiatric hospitalization included both violent and non-violent crimes.

The comparison group—which was matched to the study group by gender as indicated in the medical file as well as by the year of admission—comprised 370 male and female general psychiatric patients (GPPs), with a diagnosis of SSD according to ICD-10 (F2x) or ICD-9 (295.x) (29, 30), who had been in inpatient treatment at the Center for Integrative Psychiatry of the University Hospital of Psychiatry Zurich. This institution focuses on the sub-acute treatment of chronically ill patients with established initial pharmacotherapeutic treatment, making them a population quite suitable for comparison with FPP, who are mostly admitted from a custodial setting in which they too have received initial antipsychotic treatment.

Both facilities have secured and open wards, though, given the legal context of their institutionalization, the forensic psychiatric patients were partially and temporarily subjected to a more tightly secured treatment setting, starting in high-security wards and progressing to progressively more open wards if improving clinically.

### 2.2. Defining the outcome variable

The outcome variable (y) “forensic psychiatric patient (FPP)” was dichotomized (true vs. untrue), with “FPP – untrue” being defined as the positive class in further analysis.

### 2.3. Defining the predictor variables and data extraction

All of our data came from the case files of the patients, which included professionally documented medical histories, psychiatric and psychological reports, inpatient and outpatient reports of both hospitalizations and outpatient treatments, extensive interdisciplinary progress reports, and—for the forensic psychiatric population—testimonies, court proceedings, and data regarding previous imprisonments and detentions. The retrospective data assessment was performed independently by two experienced psychiatrists applying a directed qualitative content analysis as described by Hsieh and Shannon (32).

As part of the aforementioned ongoing overarching research project, over 500 variables were extracted (for an extensive list of all variables, including definitions, please refer to the data availability statement). As we wanted to focus on key differences related to the underlying psychiatric disorder, we selected 194 illness-related variables out of all of these variables for this analysis. A list of those variables included in the further analysis is provided in the Appendix. For a specific definition of the predictor variables, please refer to the coding protocol provided in the data availability statement.

### 2.4. Data analysis

**Figure 1:**
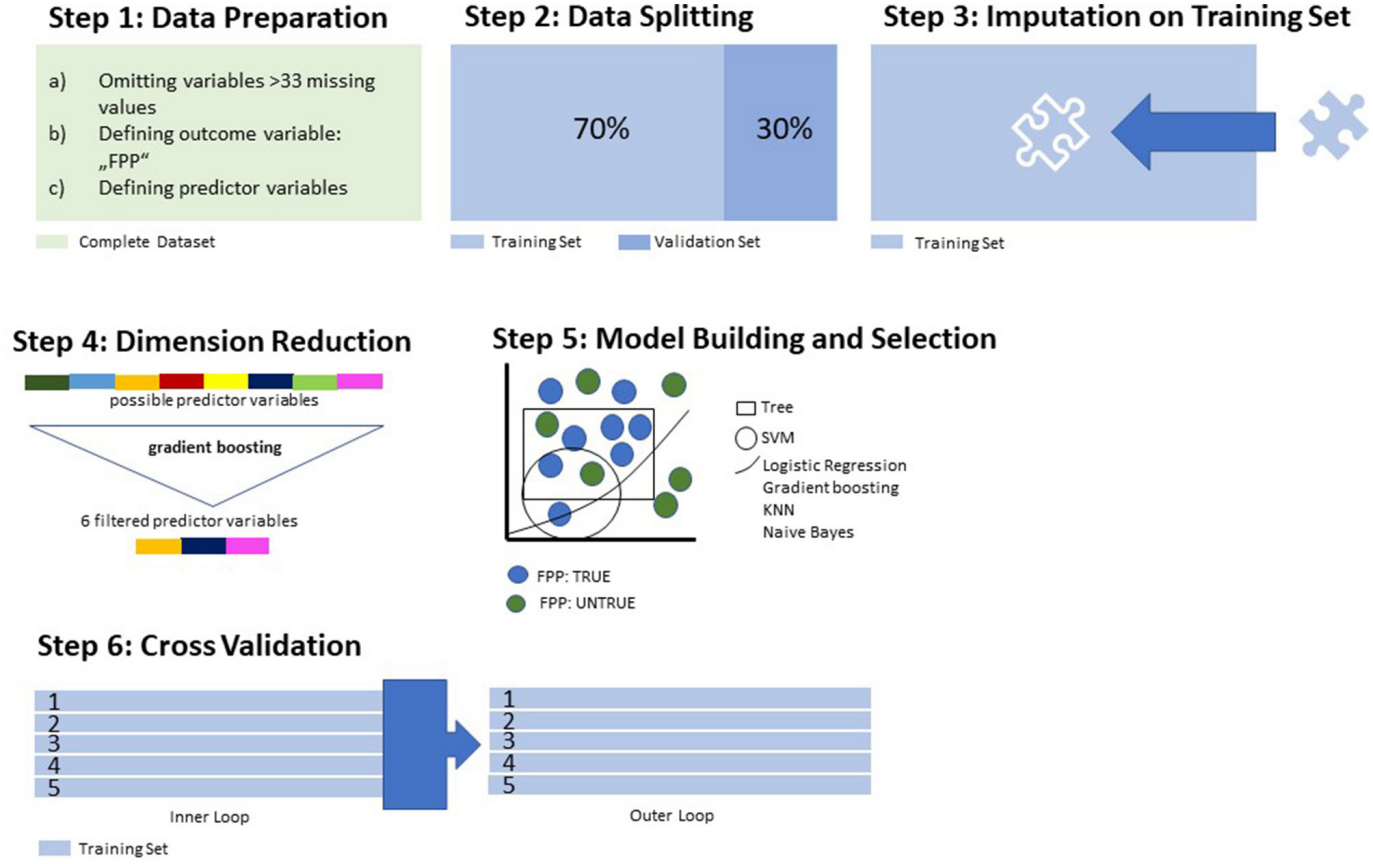
Data processing and model training.

**Figure 2:**
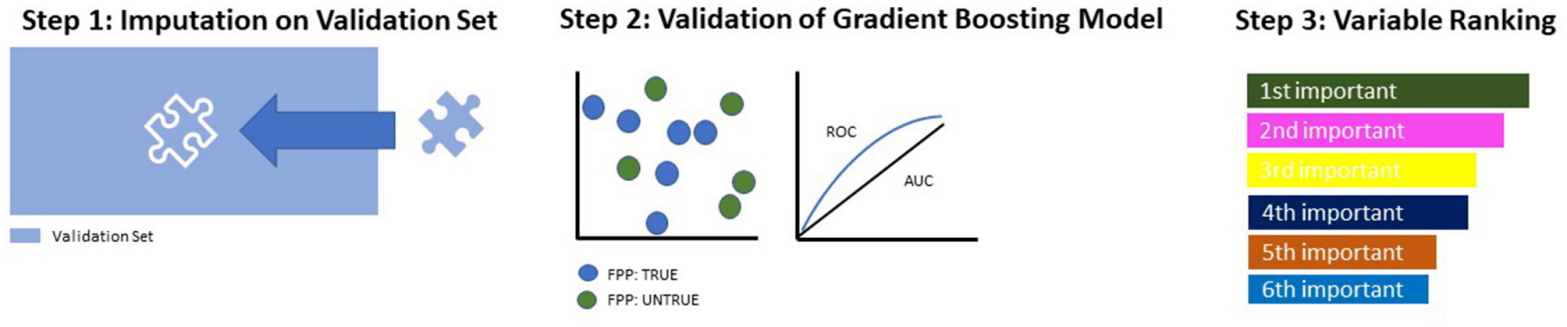
Model building and testing on the validation set.

As described in the Section 1, we aimed to exploratively identify the most indicative predictors capable of discriminating between forensic and general psychiatric patients with SSD. Thus, we chose a supervised machine learning (ML) approach for the statistical analysis. Figures 1, 2 navigate through the statistical process, which is described in further detail below.

In an initial step, all data underwent preprocessing for ML purposes (Figure 1, Step 1): Variables with >33% missing values in the total population were eliminated from further analysis. This was performed to reduce the negative influence of missing variables on the accuracy of the model (33). Categorical variables were converted to binary code. As described in Section 2.2, our outcome variable (y) was defined as “forensic psychiatric patient – true/untrue”. The dataset was then divided into two subsets: the training subset, comprising 70% of all cases, and the validation subset with the remaining 30% (Figure 1, Step 2). The validation subset was set aside for later application of the trained algorithm, which underwent the following learning process (Steps 3–5) strictly on the training subset. This step was crucial to providing the algorithms with an optimized “training ground” for the building of the model and to still have an untouched set of data for later validation purposes.

Missing values in the training set were imputed by mean for continuous variables and mode for categorical variables, as provided by the features of the MLR package (Figure 1, Step 3). The coefficients used in the imputation process were also stored for later application in the imputation process on the validation set. To spare computational resources and increase the overall performance of the model, we performed a dimension reduction through the random forest algorithm (randomForestSRC package implemented in the MLR package, evaluating variable importance) (Figure 1, Step 4). This dimensionality reduction was performed up to the point where the AUC did not improve by >5% when adding another variable. After the completion of the preprocessing features, seven different algorithms were applied to the training set for discriminative model building: logistic regression, trees, random forest, gradient boosting, k-nearest neighbor (KNN), support vector machines (SVMs), and naïve Bayes as an easily applicable generative model. These algorithms were assessed in terms of their balanced accuracy (the average of the true-positive and true-negative rates) and goodness of fit (measured with the receiver operating characteristic, balanced curve area under the curve method, ROC-balanced AUC) (Figure 1, Step 5). The model with the highest AUC— the algorithm most suitable for the data structure—was selected for the final model validation. In the next step, we aimed to reduce a common obstacle in ML, which is so-called “overfitting”. Broadly speaking, overfitting occurs when a model learns noise in the training data to such an extent that the predictive ability of the model is compromised (34, 35). To counteract this phenomenon, we performed a nested resampling. Data processing and model training were performed while embedded in cross-validation, and the performance of these models was tested in an outer loop also embedded in cross-validation. Thus, we artificially created different subsamples of the same dataset, all while the validation subset remained untouched (Figure 1, Step 6). In doing so, we averaged the error estimation over all six trials to get the total effectiveness of the model. This completed the learning process. Further steps were performed on the validation subset previously stored aside (Figure 2).

On the validation set, missing values were imputed with the same weights as on the training set, which had been previously stored (Figure 2, Step 1). Then, the gradient boosting model, earlier identified as most suitable to our data, was applied to the validation set and evaluated in terms of its ROC parameters (Figure 2, Step 2). As a final step, the variables constituting the model were ranked according to their indicative power (Figure 2, Step 3).

## 3. Results

### 3.1. Descriptive data

Patients in both groups were predominantly male (>90%), in their mid-30s, and single at the time of their admission to the referenced hospitalization. Compared to FPPs, GPPs were born in Switzerland more often. Regarding diagnoses, almost three-quarters of cases in both groups had been diagnosed with schizophrenia (F20.x according to ICD-10), while other diagnoses from the psychosis spectrum, for example, schizoaffective disorder, were less prevalent. The majority of patients also had some kind of substance use disorder as a comorbidity, with a higher prevalence among FPP. Comorbid personality disorders, while in general relatively uncommon, were also more frequently diagnosed in the FPP group. Table 1 provides an overview of these basic characteristics in both groups.

**Table 1:** Basic characteristics of the study population.

	**FPP *n/N* (%)**	**GPP *n/N* (%)**
Age at admission (mean, SD)	34.2 (10.2)	36.2 (12.2)
Sex: male	339/370 (91.6)	339/370 (91.6)
Country of birth: Switzerland	167/370 (45.1)	245/367 (66.8)
Single (at the time of admission to the referenced hospitalization)	297/364 (81.6)	282/364 (77.5)
Diagnosis: schizophrenia	294/370 (79.5)	287/370 (77.6)
Co-diagnosis: substance use disorder	269/370 (72.7)	183/327 (56)
Co-diagnosis: personality disorder	47/370 (12.7)	26/370 (7)

FPP, forensic psychiatric patients; GPP, general psychiatric patients; SD, standard deviation; N, total study population; n, subgroup with characteristics.

### 3.2. Model calculation and performance measures

Out of all of the seven algorithms applied in the learning process, gradient boosting emerged as the one with the best performance parameters on the training set, yielding a balanced accuracy of 78.5% and an AUC of 0.88 (Table 2).

**Table 2:** Machine learning models and performance in nested cross-validation.

**Statistical procedure**	**Balanced accuracy (%)**	**AUC**	**Sensitivity (%)**	**Specificity (%)**	**PPV (%)**	**NPV (%)**
Logistic regression	76.1	0.83	79.3	73	73.2	79.1
Tree	76.1	0.83	72.8	79.4	77.2	75.6
Random forest	75.4	0.86	78.9	71.9	72.5	78.6
**Gradient boosting**	**78.5**	**0.88**	**76.6**	**80.4**	**78.4**	**78.3**
KNN	74.5	0.81	81.7	67.3	70.1	80.6
SVM	73.5	0.82	74.3	72.6	71.5	74.9
Naive Bayes	75.9	0.83	78.4	73.4	73.3	78.4

AUC, area under the curve (level of discrimination); PPV, positive predictive value; NPV, negative predictive value; KNN, k-nearest neighbors; SVM, support vector machines. “FPP – untrue” was defined as a positive class. Bold blue font indicates the algorithm with the best performance parameters.

After the reduction in dimensionality through the application of random forest down to the point where the AUC of the model did not improve by >5% when adding another variable, six variables emerged as most discriminative regarding the outcome variable (see Table 3).

**Table 3:** Distribution of predictor variables after dimensionality reduction.

Variable code^*^	**Variable description**	**FPP N/%**	**GPP N/%**
PH18a	Outpatient psychiatric treatment(s) before referenced hospitalization	179/340 (52.6)	**275/326 (84.4)**
PH23a	Neuroleptic medication in psychiatric history	224/370 (60.5)	**330/353 (93.5)**
PH23p	History of medication compliance	23/204 (11.3)	**166/304 (54.6)**
PH24a	Any other type of pharmacotherapy in psychiatric history^*a*^	159/229 (69.4)	**254/301 (84.4)**
R9e	Olanzapine equivalent at discharge from ref. hospitalization (in mg, mean, and SD)	**22.1 (12.3)**	19.3 (14.2)
R13a	Compulsory measure during referenced hospitalization	**131/358 (36.6)**	51/353 (14.4)

Bold font indicates the group with the higher expression of each item. N, total study population; n, subgroup with characteristic; SD, standard deviation. ^*^For a detailed definition of each of these variables, please refer to the coding sheet provided in the data availability statement. ^*a*^refers to any type of psychiatric medication other than antipsychotic medication. Herbal and homeopathic remedies are not considered in this item.

### 3.3. Final model performance

Applied to the validation subset (30% of all cases), gradient boosting was performed with a balanced accuracy of 77.5% and an AUC of 0.86; it can therefore be considered that the model features an excellent ability to distinguish between the two groups (36). Both patients with forensic and general psychiatric backgrounds were correctly identified in most cases, as indicated by a sensitivity of 71.4% and a specificity of 83.5% (see Table 4).

**Table 4:** Performance measures of gradient boosting on the validation subset.

**Performance measures**	**% (95% CI)**
Balanced accuracy	77.5 (71.4–82.2)
AUC	0.86 (0.81–0.91)
Sensitivity	71.4 (62.3–79.1)
Specificity	83.5 (74.6–89.8)
PPV	83.3 (74.4–89.7)
NPV	71.7 (62.6–79.3)

CI, confidence interval; AUC, area under the curve (level of discrimination); sensitivity, true positive/(true positive + false negative); specificity, true negative/true negative + false positive; PPV, positive predictive value; NPV, negative predictive value.

### 3.4. Ranking of predictor variables

**Figure 3:**
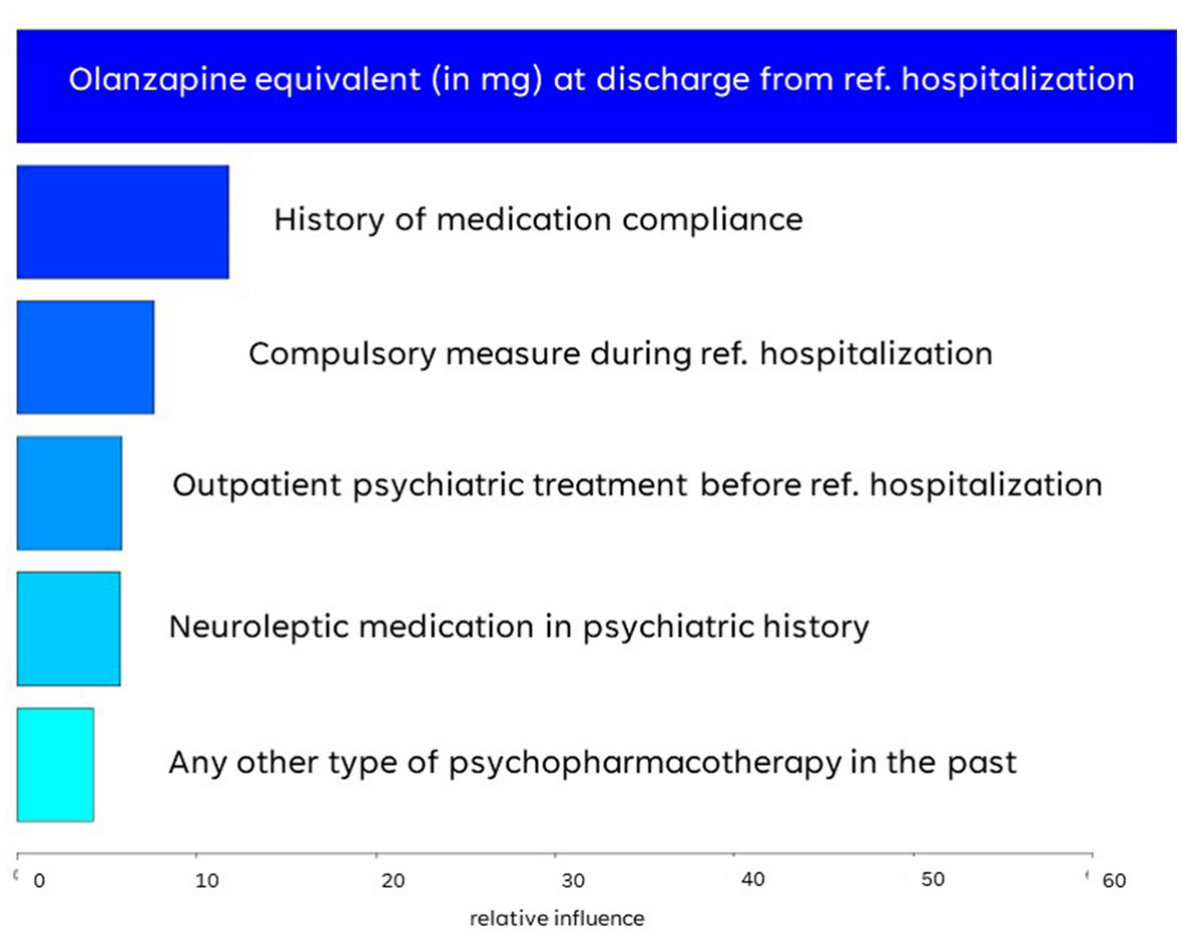
Variable importance ranked by gradient boosting algorithm.

In the ranking of their contribution to the model, the olanzapine equivalent upon discharge emerged as the most indicative, whereas the remaining five variables had a similar influence (see one-sided tornado graph, Figure 3).

## 4. Discussion

While certain risk factors for offending as a negative outcome of a mental disorder are well-established and reproducible, there is still uncertainty and inconsistency about which patients affected by SSDs tend to perform some kind of illegal act during the course of their illness. The purpose of this study was, therefore, to evaluate clinical key factors distinguishing offenders and non-offenders with SSD based on items regarding the underlying mental illness, using supervised machine learning (ML) as a statistical approach with the ability to analyze the interplay of a large quantity of potentially influential variables. Out of 194 possible predictor variables, six emerged as defining a gradient boosting model yielding a balanced accuracy of 77.5% and an AUC of 0.86.

The cumulative antipsychotic dose, defined by the prescribed olanzapine equivalent upon discharge from the referenced hospitalization, emerged as a variable with by far the highest relative influence: With a mean dose of 22 mg, FPP had higher antipsychotic doses when released from the institution than their GPP counterparts (mean dose: 19 mg). While the literature on prescription patterns in forensic psychiatric settings is sparse, higher doses of antipsychotics in offender populations in comparison with the general population have been sporadically reported by other authors: Stone-Brown et al. (37) described a high-dose antipsychotic rate of ~30% in a mixed-diagnosis prison population (37). In an Italian forensic psychiatric sample, also with diagnostic heterogeneity, high-dose antipsychotics were even administered in around half the cases (38). In contrast, in general psychiatry, high-dose antipsychotic rates of ~20% are reported, although generally, there is a lack of consensus as to how exactly high-dose prescription is defined, thus making a comparison between studies difficult (39–42). There are several possible explanations for these differences in dosing between general and forensic psychiatric populations. It has been previously suggested that the prescription patterns in offender populations with mental health problems are not merely, maybe not even primarily, driven by illness-related aspects. For instance, Mandarelli et al. (38) reported an association between aggressive behavior and higher doses of antipsychotics but could find no diagnostic correlation (38). They hypothesized that pharmacotherapeutic interventions are one of the few treatment options available in a forensic psychiatric setting in which there is oftentimes, especially in the initial treatment phase, a lack of cooperation and insights into the necessity of treatment on the patient's side. With the forensic psychiatrist's responsibility to not only tend to the individual needs of the patient but to reduce their social dangerousness (fulfill legal obligations/impositions) and prevent illness-driven reoffending, an intensified pharmacotherapeutic approach may be the result of said double-mandate (43, 44). That being said, it has to be noted that, although plasma levels of antipsychotic medication were regularly obtained in the FPP sample, the retrospective data may not sufficiently illustrate whether the patient was regularly taking the medication prescribed.

At the same time, GPPs were characterized by a higher rate of prescriptions of other psychopharmacologic substances, e.g., benzodiazepines and antidepressants, than FPP. This is highly contradictory to an Italian prison study on offenders with mental illness, which observed that psychopharmacologic agents additional to antipsychotics were administered in even 92% of cases (45). A small-sample UK prison study reported concurrent psychopharmacotherapies in 85% of cases (46). However, both the UK and the Italian study populations were quite heterogenous in their diagnoses, and there was a high rate of off-label prescriptions common in prison settings, such as anxiolytics and hypnotics (46, 47). Due to the considerable diagnostic differences, robust comparability of our results is hardly given. Yet, it does, indeed, seem contradictory that GPPs were more likely to receive additional sedatives than FPPs, even though the latter can be considered to be at higher risk of impulsivity and agitation. The authors have previously suggested that the already high doses of antipsychotics in FPP could have made further sedation, i.e., with benzodiazepines, unnecessary. Another possible explanation may lie in the concern of a paradoxical reaction to hypnotics, which—although generally rare at <1%—are more likely in patients with a history of aggressive behavior (48). Further research on prescription patterns in the forensic psychiatric treatment of patients with SSD is necessary to evaluate these hypotheses before drawing clinical conclusions.

FPPs were less likely to have had outpatient treatment and a prescription for antipsychotic medication before the referenced hospitalization than their GPP comparison group. Insufficient embedment into the mental healthcare system and thus insufficient treatment is a known correlate of criminal behavior as a negative outcome during the course of SSD (49). Previous research has also demonstrated a particularly high risk of offending during the first psychotic episode, with untreated impulsivity as a major mediator of aggressiveness toward others, but also toward oneself. Nielssen et al. (50) reported that over a third of all fatal and non-fatal serious assaults committed by patients with SSD occurred during an untreated first psychotic episode (50). Poor executive functioning and poor self-care skills—both also associated with violence in psychosis—may further hinder affected individuals from seeking help (51).

FPP also had a much lower rate of pharmacotherapeutic compliance (11% vs. 55%). This finding supports the previous literature, according to which violent and non-violent offenses are associated with lower levels of treatment adherence (or vice versa) (37, 52–54). Intuitively, one would assume that lower levels of insight into the underlying mental illness within forensic psychiatric populations are responsible for the reported lack of adherence. A lack of insight into their mental illness and subsequently into the need for treatment is common for individuals with SSD, especially with active symptoms, as the perception of the surroundings and one's self are altered due to the underlying pathology (55). However, forensic psychiatric patients with SSD appear to be similarly aware of their condition as general psychiatric patients, as demonstrated by an Australian study (56). Alia-Klein et al. (57) argued that a binary definition fails to cover the complexity of the phenomenon of insight into illness (57). In a forensic psychiatric sample of patients suffering from psychosis, they found that non-adherence was, in fact, independent of poor insights. Instead, they suggested that patients could be cognitively aware of their mental disorder, but, at the same time, lack affective concern regarding it. In their evaluation of offenders with schizophrenia, Rezansoff et al. (54) too argued against the binary definition of treatment compliance and found an increased risk of violent and non-violent offenses in individuals with lower levels of compliance (54). Authors investigating the construct of awareness and compliance in general psychiatric populations, such as Dam et al. (58), have also argued that a dichotomous definition fails to cover the many layers of insight and compliance and that the two are certainly interwoven but cannot be assumed to be synonymous (58). It seems, therefore, worthwhile to further explore factors driving (non-)adherence in forensic psychiatric samples.

FPP proved to have a higher rate of compulsory measures during the referenced hospitalization, such as mechanical restraint, isolation/seclusion, or compulsory application of medication. In Switzerland, coercive measures are taken as a last resort either in emergency situations, i.e., to avert immediate danger to oneself or others, or electively in the case of patients who are incapable of giving consent and are no longer able to make a decision themselves (59, 60). One hypothesis for the higher rate of compulsory measures in the FPP sample is the higher prevalence of self-harm in offender populations (61). The more frequent use of coercion in FPP is also unsurprising given that violence or threat of violence—with a high prevalence in FPP populations—is among the most frequently identified factors associated with coercive measures (62–65). As this parameter reflects coercive measures during forensic hospitalization, thus, after the committed offense, clinicians may also be quicker to opt for coercive measures if the patient has been deemed particularly dangerous. This may be especially the case for patients whose behavior is closely linked to the dynamics of their offense. At the same time, reported rates of coercive measures show a wide range both in forensic and general psychiatry, and a higher prevalence of such interventions has not been well-established for forensic psychiatric populations (66). This research gap results from a lack of coherent definitions of coercion and different research methods altogether. Thus, empirical research on coercive measures in forensic psychiatry is scarce and needs to be expanded further, especially since ethical aspects are stressed in the forensic psychiatric context with already restricted treatment settings.

Strikingly, all of the six most distinguishing items related to treatment, e.g., the severity of psychopathology, type of symptoms, or comorbidities such as substance use and personality disorders did not emerge as dominant in the model. This seems surprising at first glance; after all, a higher prevalence of substance use disorders and certain personality disorders in offender populations than in the general population has been described by several previous research groups (67–70). The comorbidities are also known risk factors for violent behavior, which also makes a consequential involvement with the criminal justice system more likely (20, 71). The same goes for the prevalence of certain symptoms of SSD, which are considered to increase the likelihood of offending, such as a higher expression of positive symptoms and general severity of symptoms—although there is still controversy over which types of symptoms exactly promote criminal behavior in SSD, and corresponding findings are oftentimes inconsistent (72, 73). The fact that our findings diverge from these previous results by no means indicates that known and well-established risk factors for problematic and potentially illegal behavior are contradicted. Psychopathology, for instance, might be featured indirectly in the model, i.e., through the items regarding psychopharmacologic prescriptions and higher doses. Furthermore, with the forensic psychiatric institution being much more structured and restrictive than the general psychiatric facility, the setting of care may have influenced the outcome regarding the expressiveness and influence of psychopathology. Nevertheless, while a certain indirect influence of said domains seems likely and cannot by any means be ruled out entirely, our findings suggest that criminal development does not merely depend on the presence of unfavorable symptomatology and morbidity. In fact, it seems as if the likelihood of offense as a negative outcome can be meaningfully reduced if the affected patient is well integrated into the mental healthcare system and adequately and sufficiently treated. This aspect of compensability of certain traits as well-established stand-alone risk factors for violence and offending has already been discussed in light of previous results of the authors regarding aggression as the negative outcome of an SSD but has not been widely covered in other existing literature (44). The crime-preventive strength of a sufficient embedment in the mental healthcare and support system emphasizes the importance of early-on detection of mental disorders and potential comorbidities and the proper integration of the affected individual into appropriate and available structures. In this context, clinicians need to make an effort to promote understanding and awareness of individual needs, risk factors, and protective resources that could be strengthened.

### 4.1. Strengths and limitations

Regarding the potential limitations of the presented study, the retrospective design needs to be addressed. Retrospective designs are considered inferior to prospective studies for numerous reasons. First of all, the quality of the data depends highly on the quality and accuracy of the documentation (74). This can be especially problematic for variables that lack a clear definition, e.g., “negative attitude toward staff”, which may be interpreted differently when assessed by different clinicians. Furthermore, as a common obstacle in retrospective designs, some variables had a high rate of missing values and had to be excluded from further analysis. Thus, there is a certain risk of bias from the resources in this study. One could also argue that, with the development of, for example, other pharmacological agents, the broad timeframe for case inclusion (spanning over 30 years) could skew the results in some respects. However, with the duration of inpatient forensic treatment in Switzerland being rather long (Article 59 foresees 5 years of treatment, which may be prolonged), a shorter timeframe would have led to a much smaller sample, thus reducing the robustness of the results. Regarding statistical limitations, one has to be aware that while the dataset is rather larger from a forensic psychiatric point of view, it is merely moderate for ML purposes. The larger the dataset, the better the performance of an ML algorithm. As a result, our model is more likely to be subjected to overfitting than an even larger sample would be—a risk we have attempted to counteract through the application of nested cross-validation (35). However, while the cross-validation limits overfitting, it does not completely solve the issue—although with the small delta in performance parameters between the training and validation sets, the effect of cross-validation appears to be significant. Again, a training process on an even larger sample would be an advisable approach for future research. Less as a limitation and more as a fact that should be kept in mind when interpreting the results, possible multicollinearity has to be considered, meaning that items that did not emerge as most indicative in the gradient boosting model could have had an indirect influence as well. For instance, the higher dose of antipsychotics in the FPP group may have been the result of a higher prevalence of aggressive behavior or a more severe degree of symptomatology. Another important note is that the mediators for violent vs. non-violent crime may be different for individuals with SSD—as both types of offenders were included in this study, no discrimination in that regard can be made based on our results. Finally, since our population consisted of male patients in over 90% of cases, it is not possible to derive implications for female populations from our results. The same goes for differentiation between forensic and general psychiatric outpatients, as we only focused on patients in inpatient settings. Yet, we opted to include the small available number of women to represent a patient population that corresponds to the reality of the penitentiary system (which, with that in mind, could be considered a strength of our study). Further strengths lie in the study's ability to close a significant research gap: Comparative studies on forensic psychiatric patients with SSD are scarce, even though this population can be considered highly relevant due to its well-established elevated risk of aggressive and violent behavior (11, 75). With a total of 740 rather homogenous cases, this is—to the authors' knowledge—one of the largest comparative analyses of said group. Furthermore, the application of ML allowed the analysis of a large number of variables as well as their interplay in a multidimensional model, while most commonly used statistical procedures are limited in that regard (e.g., null-hypothesis significance testing) (76–78).

## 5. Conclusion

In summary, the study presented sheds light on factors distinguishing individuals with SSD who end up involved with the judicial system from those who do not. Forensic psychiatric patients showed worse integration into outpatient treatment facilities, a lower ratio of medication compliance, and prescriptions of antipsychotics as well as other psychopharmacotherapies substances but a higher mean antipsychotic dose and a higher likelihood of undergoing coercive measures during hospitalizations than their comparison group from general psychiatry. At the same time, the domain of psychopathology does not seem to be a major distinguishing factor between the two groups. Through the application of artificial intelligence, the complex interplay of risk and protective factors in the development of criminal behavior in individuals with SSD could be further explored. Without a profound understanding of both, the establishment of effective preventive measures is not possible. While this research gap, which is well in need of closing, will remain unless the results are reproduced, validated, and thus proven robust in larger populations, our findings promote, once again, the efforts of clinical and health political agents to integrate individuals affected by SSD into the mental healthcare system. This advocation includes low financial and administrative barriers for entering therapeutic institutions, for both in- and out-patients.

## Data availability statement

The datasets presented in this study can be found in online repositories. The names of the repository/repositories and accession number(s) can be found at: https://www.researchgate.net/publication/363044110_Coding_protocol_Pathways_into_delinquency_in_offenders_suffering_from_schizophrenia_spectrum_disorders.

## Ethics statement

The studies involving humans were approved by the Ethikkommission des Kantons Zürich (KEK Zürich), Switzerland. The studies were conducted in accordance with local legislation and institutional requirements. The Ethics Committee/institutional review board waived the requirement of written informed consent for participation from the participants or the participants' legal guardians/next of kin because this research project was reviewed and approved by the Ethics Committee Zurich under the reference number KEK-ZH-NR 2014-0480. Patient consent was waived due to the retrospective design, for which formal consent is not necessarily required in the Canton of Zurich.

## Author contributions

LM and JK: conceptualization, methodology, validation, formal analysis, investigation, and data curation. JK: software, resources, supervision, and project administration. LM: writing—original draft preparation and visualization. LM, AH, JM, and JK: writing—reviewing and editing. All authors contributed to the article and approved the submitted version.
