# Supplementary material for: Offenders and non-offenders with schizophrenia spectrum disorders: the crime-preventive potential of sufficient embedment in the mental healthcare and support system
Source: Front Psychiatry. 2023 Aug 30;14:1231851. doi: 10.3389/fpsyt.2023.1231851 (PMC10498463; doi:10.3389/fpsyt.2023.1231851)
Supplement: Supplementary file 1 [file Table_1.pdf]

1 Appendix

**Variable Code**	**Label**	**Definition**
PH1	Age at which the F2x diagnosis was given?	Age (in years) at which the patient was diagnosed with any schizophrenia spectrum disorder for the first time (ICD-10: F2x.x)
PH2	Age at which the patient showed first symptoms of the F2x diagnosis?	Age (in years) at which he/she showed any of the symptoms that are described in the ICD-10 under the corresponding F2x.x diagnosis (see item PH1) for the first time; **unless** the symptoms can be better explained by other causes (e. g. substance consumption)
PH3	History of delusions	Yes, if he/she (had) experienced any type of delusion (persecutory delusions, delusions of grandeur). 'Delusion' was defined as ''a false belief based on incorrect inference about external reality that is firmly sustained despite what almost everybody else believes and despite what constitutes incontrovertible and obvious proof or evidence to the contrary. The belief is not one ordinarily accepted by other members of the person's culture or subculture (e.g. it is not an article of religious faith)''
PH4	History of hallucinations	Yes, if he/she (had) experienced any visual, auditory, olfactory, tactile and/or gustatory hallucinations.
PH5	History of penetrability of the own ego	Yes, if he/she (had) experienced any of the following: thought insertion, thought withdrawal, thought broadcasting, thought echo, depersonalisation, derealisation and/or delusions of control
PH6	History of disorders of affect or drive	Yes, if he/she (had) experienced any mania- and/or depression-like symptoms with the consequence of any type of functional impairment (distress and/or disability)
PH7	History of negative symptoms	Yes, if he/she (had) experienced any types of negative symptoms which are listed in the positive and negative syndrome scale (PANSS), which cannot be better explained by other causes (such as, for example, another mental illness)
PH10a	History of suicide attempts	Yes, if he/she had harmed himself/herself intentionally with suicidal intent at any one time before the investigated offence respectively the time of the referenced hospitalization
PH11a	History of endangerment of others	Yes, if, at any one time before the investigated offence respectively the referenced hospitalization, he/she had deliberately or negligently put one or more other person(s) at any type of risk (e.g., by the exertion of violence), which entailed the potential or actual consequence of a substantial mental and/or physical impairment of the corresponding person(s)
PH12a	Was there ever any compulsory measure during a hospitalization used?	Yes, if one or more of the following measures had been forced upon the patient in a psychiatric hospital at any one time before the investigated offence respectively the referenced hospitalization:<br>- involuntary isolation<br>- mechanical fixation (any type)<br>- compulsory medication (oral AND/OR parenteral)<br>- involuntary retention in the clinic (after voluntary admission)

PH13	Alcohol abuse	Yes, if he/she (had) had an alcohol consumption pattern corresponding to the ICD-10 diagnosis ‘‘Harmful use (F10.1)’’ or ‘‘Dependence syndrome (F10.2x)’’ at the time of or at any one time before the investigated offence respectively the referenced hospitalization
PH14a	No drug abuse	Yes, if he/she did not have and had not had a substance consumption pattern corresponding to the ICD-10 diagnosis ‘‘Harmful use (F1x.1)’’ or ‘‘Dependence syndrome (F1x.2x)’’ at the time of and at any one time before the investigated offence respectively the referenced hospitalization. In this item, the term ‘substance’ refers to illicit drugs and prescription medications but **not** to alcohol.
PH14b	Cannabis abuse/-dependence?	Yes, if he/she (had) had a cannabinoid consumption pattern corresponding to the ICD-10 diagnosis ‘‘Harmful use (F12.1)’’ or ‘’Dependence syndrome (F12.2x)’’ at the time of or at any one time before the investigated offence respectively the referenced hospitalization
PH14c	Opioid abuse/-dependence?	Yes, if he/she (had) had an opioid consumption pattern corresponding to the ICD-10 diagnosis ‘‘Harmful use (F11.1)’’ or ‘‘Dependence syndrome (F11.2x)’’ at the time of or at any one time before the investigated offence respectively the referenced hospitalization
PH14d	Cocaine abuse/-dependence?	Yes, if he/she (had) had a cocaine consumption pattern corresponding to the ICD-10 diagnosis ‘‘Harmful use (F14.1)’’ or ‘‘Dependence syndrome (F14.2x)’’ at the time of or at any time before the investigated offence respectively the referenced hospitalization
PH14e	consumption of stimulants, amphetamines, ecstasy?	Yes, if he/she (had) had a stimulant consumption pattern corresponding to the ICD-10 diagnosis ‘‘Harmful use (F15.1)’’ or ‘’Dependence syndrome (F15.2x)’’ at the time of or at any time before the investigated offence respectively the referenced hospitalization; except for cocaine (see item PH14d) and caffeine
PH15a	Presence of a personality disorder (PD)?	Yes, if he/she had ever been diagnosed with any personality disorder listed in the ICD-10 (F60.x, F61) and/or the DSM-V
PH16a	Personality disorder: paranoid	Yes, if he/she was diagnosed with paranoid personality disorder and/or exhibited a stable pattern of symptoms that can be considered compatible with a paranoid personality disorder (ICD-10: F60.0) or a paranoid accentuation of personality traits (ICD-10: Z73.1) at the time of or at any time before the investigated offence respectively the referenced hospitalization
PH16b	Personality disorder: schizoid	Yes, if he/she was diagnosed with schizoid personality disorder and/or exhibited a stable pattern of symptoms that can be considered compatible with a schizoid personality disorder (ICD-10: F60.1) or a schizoid accentuation of personality traits (ICD-10: Z73.1) at the time of or at any time before the investigated offence respectively the referenced hospitalization
PH16c	Personality disorder: dissocial	Yes, if he/she was diagnosed with dissocial or antisocial personality disorder and/or exhibited a stable pattern of symptoms that can be considered compatible with a dissocial personality disorder (ICD-10: F60.2) or a dissocial accentuation of personality traits (ICD-10: Z73.1) at the time of or at any time before the investigated offence respectively the referenced hospitalization

PH16d	Personality disorder: emotionally unstable	Yes, if he/she was diagnosed with emotionally unstable or borderline personality disorder and/or exhibited a stable pattern of symptoms that can be considered compatible with an emotionally unstable personality disorder (ICD-10: F60.3x) or an emotionally unstable accentuation of personality traits (ICD-10: Z73.1) at the time of or at any time before the investigated offence respectively the referenced hospitalization
PH16e	Personality disorder: anancastic	Yes, if he/she was diagnosed with anancastic or obsessive-compulsive personality disorder and/or exhibited a stable pattern of symptoms that can be considered compatible with an anancastic personality disorder (ICD-10: F60.5) or an anancastic accentuation of personality traits (ICD-10: Z73.1) at the time of or at any time before the investigated offence respectively the referenced hospitalization
PH16f	Personality disorder: dependent	Yes, if he/she was diagnosed with dependent personality disorder and/or exhibited a stable pattern of symptoms that can be considered compatible with a dependent personality disorder (ICD-10: F60.7) or a dependent accentuation of personality traits (ICD-10: Z73.1) at the time of or at any time before the investigated offence respectively the referenced hospitalization
PH16g	Personality disorder: histrionic	Yes, if he/she was diagnosed with histrionic personality disorder and/or exhibited a stable pattern of symptoms that can be considered compatible with a histrionic personality disorder (ICD-10: F60.4) or a histrionic accentuation of personality traits (ICD-10: Z73.1) at the time of or at any time before the investigated offence respectively the referenced hospitalization
PH16h	Personality disorder: narcissistic	Yes, if he/she was diagnosed with narcissistic personality disorder and/or exhibited a stable pattern of symptoms that can be considered compatible with a narcissistic personality disorder (ICD-10: F60.8) or a narcissistic accentuation of personality traits (ICD-10: Z73.1) at the time of or at any time before the investigated offence respectively the referenced hospitalization
PH17a	Any other psychiatric comorbidity	Yes, if he/she was diagnosed with any mental illness listed in the ICD-10 or DSM-5 at the time of the investigated offence except for the ones covered in the aforementioned items
PH18a	Any outpatient psychiatric treatment(s)?	Yes, if he/she had visited a mental health care provider (psychologist and/or psychiatrist) as an outpatient at any time before the investigated offence respectively the referenced hospitalization, regardless of the duration of said treatment
PH19a	Any inpatient psychiatric treatment(s)?	Yes, if he/she had been an inpatient and/or a semi-inpatient in a mental health care institution at any time before the investigated offence respectively the referenced hospitalization, regardless of the duration of said treatment
PH22a	Was the patient ever compulsorily admitted?	Yes, if he/she had been involuntarily admitted to any mental health care institution by order of an authorised person (such as a physician) at any one time before the investigated offence respectively the referenced hospitalization. Compulsory admission is defined as application of the Swiss “Fürsorgerische Unterbringung (FU)” OR “Fürsorgerischer Freiheitsentzug (FFE)” according to the Swiss child and adult protection law (KESR).

PH23a	Antipsychotic medication	Yes, if he/she had been prescribed any antipsychotic medication at any time before the investigated offence respectively the referenced hospitalization<br><br>(PH23b-o are covering all substances separately)
PH23p	Regular intake of antipsychotic medication	Yes, if he/she, mental health professionals and trusted private persons (e.g., close family members) had **not** reported/documented a lack of compliance/adherence to any antipsychotic medications at any time before the investigated offence **AND** if mental health professionals and trusted private persons (e.g., close family members) had **not** had reasonable grounds for suspecting that the patient lacked medication compliance/adherence to any antipsychotic medications at any time before the investigated offence respectively the referenced hospitalization.
PH24a	Any other type of pharmacotherapy in psychiatric history	Yes, if he/she had been prescribed any type of psychiatric medication at any time before the investigated offence respectively the referenced hospitalization; except for antipsychotic medications<br><br>(PH24b-e are covering the substance groups separately)
PH25a	Any problems in the treatment of the patient before the investigated offence / hospitalization?	Yes, if any of the following items PH25b – PH25i applies: Noncompliance, did not go to his outpatient treatment, consumed alcohol/drugs, homelessness, no health insurance, treatment for other diagnosis, non adherence to therapy/refused therapy
PH25j	If there were any difficulties (PH25a YES), was there any reaction?	Yes, if measures to counteract the problems in treatment were taken (e. g. establishment of some form of assisted living, compulsory admissions, establishment of legal supervision)
PH26a	Alerts by third parties about danger originating from the patient	Yes, if any report from a private person, an official, a public authority and/or a person involved in the individual's care had indicated a substantially increased risk for any type of delinquency originating from the individual at any time before the investigated offence resp. the referenced hospitalization (PH26b: was there any reaction)
PH27a	Legal complaints by third parties	Yes, if any private person, official, public authority and/or person involved in the individual's care had filed a police/legal complaint at any time before the investigated offence resp. the referenced hospitalization
PH27b	Efforts by third parties to seek psychiatric help	Yes, if he/she, mental health professionals or trusted private persons (e.g., close family members) had reported/documented that any relative(s) and/or legal guardian repeatedly had advised and/or urged him/her to seek psychiatric treatment at any time before the investigated offence AND/OR if he/she had been involuntarily admitted to a mental health care institution on request of any relative(s) and/or legal guardian at any time before the investigated offence resp. the ref. hospitalization
R1a-d	Current psychiatric F2x-diagnosis	Diagnosis acc. to ICD-10 given at the time of admission to the referenced forensic hospitalization
R8a-q	Pharmacotherapy during referenced hospitalization	Substances prescribed during the referenced hospitalization unless the targeted administration dose was not reached
R3	Was the patient first diagnosed at admission?	Yes, if his/her diagnosis of schizophrenia spectrum disorder (ICD-10: F2x.x) at the time of discharge from the referenced forensic hospital had been given within the first month after admission to the referenced forensic hospital

R9e	Olanzapine equivalents at the time of discharge?	Cumulative dose of antipsychotic medication included in his/her prescription schedule at the time of discharge from the referenced forensic hospital converted to olanzapine equivalents in milligrams
R9f	Polypharmacy at discharge	Yes, if his/her prescription schedule included two or more different antipsychotics at the time of discharge from the referenced hospital
R10	Was the patient ever suicidal during the referenced hospitalization?	Yes, if he/she reported that he/she has experienced active suicidal ideation at any one time during the referenced hospitalisation **AND/OR** if he/she attempted suicide during the current hospitalisation (see item R12)
R11	Did the patient ever show any self-harming behavior during the referenced hospitalization?	Yes, if he/she intentionally harmed himself/herself (by cutting, burning or other means) at any one time during the referenced hospitalization (including self-harming behavior with suicidal intent (see item R12))
R12	Did the patient ever attempt suicide during the referenced hospitalization?	Yes, if he/she intentionally harmed himself/herself with suicidal intent at any one time during the referenced hospitalization
R13a	Compulsory measure	Yes, if one or more of the following measures was forced upon him/her during the referenced forensic hospitalization:<br>- involuntary isolation<br>- mechanical fixation (any type)<br>- compulsory medication (any type)
R14	Compulsory measure as consequence of endangerment of others	Yes, if, at any one time during the referenced forensic hospitalisation, he/she deliberately or negligently put one or more other person(s) at any type of risk (e.g., by the exertion of violence) which entailed the potential or actual consequence of a substantial mental and/or physical impairment of the corresponding person(s), thus leading to a compulsory measure
R15a-g	Main content of psychotherapy during ref. hospitalization	Yes, if one of the following subjects was taken up in > 50 % of therapeutic conversations during the referenced hospitalization: regulations, daily hospital routine, biography, offending, medication, disorder/symptoms, future perspectives
R16a	No Problems during psychotherapy	Yes, if none of the items 16b – 16h is answered with ‘yes’ (refers to referenced hospitalization)
R16b-h	Type of problems during psychotherapy	Lack of compliance, break off by therapist/patient, deterioration, extensive cognitive impairment, language barrier, severe occupation with delusions
R17a	No occupational/ergo therapy	Yes, if he/she participated type in 10 or less ergo therapy sessions during the referenced forensic hospitalization
R17b-g	Type of occupational/ergo therapy	Basic work, small household task, basic industrial work, advanced industrial work, gardening, woodwork
R18	Problems during occupational/ ergo therapy	Yes, if, during the referenced hospitalization, three or more ergo therapists independently reported or indicated that the patient had shown a lack of compliance, (neuro)cognitive impairments, psychological disabilities, antisocial behavior and/or linguistic problems which had had a substantial negative impact on the course of an ergo therapy session
R20b/c	Did the patient ever show any endangerment of others during the referenced hospitalization?	Yes, if he/she showed any physical or verbal aggressions towards one or more staff member(s) or one or more other patient(s) at any one time during the referenced hospitalization.

		Note: Aggression is defined as ‘’a behavior that is intended to harm another person who Is motivated to avoid that harm’’
R20d	Damage of property during ref. hospitalization	Yes, if, at any one time during the referenced hospitalization, he/she intentionally damaged any property that was not under his/her ownership
R20e	Came back late from administrative leave during ref. hospitalization	Yes, if, at any one time during the referenced hospitalization, he/she stayed away from the station for longer than allowed but eventually returned without police
R20f	Threat/attempt to escape during ref. hospitalization	Yes, if, at any one time during the referenced hospitalization, he/she threatened and/or was reported to have attempted to leave the station without permission (regardless of whether he/she had stated that she would eventually return or not; and regardless of whether he/she actually left the station or not)
R20g	escape during ref. hospitalization	Yes, if, at any one time during the referenced hospitalization, he/she left the station without permission OR stayed away from the station for longer than allowed and was eventually returned by the police
R20h	Intake/dealing of alcohol/drugs/medication during ref. hospitalization	Yes, if, at any one time during the referenced hospitalization, mental health professional confirmed that he/she had engaged in the prohibited consumption and/or trade of substances (including alcohol and prescription medications) OR if, at any one time during the referenced hospitalization, there were reasonable grounds for suspecting that he/she had engaged in the prohibited consumption and/or trade of substances (including alcohol and prescription medications)
R20i	Theft during ref. hospitalization	Yes, if, at any one time during the referenced hospitalization, he/she was reported to have illicitly taken possession of any type of object that was not under his/her ownership
R20j	Sexual assault during ref. hospitalization	Yes, if, at any one time during the referenced hospitalization, he/she was reported to have shown any verbal aggressions, physical aggressions and/or otherwise intimidating behavior that aimed at forcing a victim to any type of unwanted sexual behavior
R27a	Evaluation of results of treatment regarding F2x-diagnosis	Estimated change in symptom severity and functional impairment (distress and/or functional impairment) during the course of the stay in the referenced hospital
R27c	Evaluation of results of treatment regarding F1x-diagnosis	Estimated change in the risk of relapsing into a substance consumption pattern corresponding to the ICD-10 diagnosis ‘’Harmful use (F10.1)’’ or ‘’Dependence syndrome (F10.2x)’’ during the course of the stay in the referenced hospital
PA1	PANSS at admission: Scale Delusion	Score in the PANSS item ‘delusion’ at the time of admission to the referenced hospital
PA2	PANSS at admission: Scale Conceptual disorganization	Score in the PANSS item ‘conceptual disorganization’ at the time of admission to the referenced hospital

PA3	PANSS at admission: Scale Hallucinations	Score in the PANSS item ‘hallucinations’ at the time of admission to the referenced hospital
PA4	PANSS at admission: Scale Hyperactivity	Score in the PANSS item ‘hyperactivity’ at the time of admission to the referenced hospital
PA5	PANSS at admission: Scale Grandiosity	Score in the PANSS item ‘grandiosity’ at the time of admission to the referenced hospital
PA6	PANSS at admission: Scale suspiciousness/persecution	Score in the PANSS item ‘suspiciousness/persecution’ at the time of admission to the referenced hospital
PA7	PANSS at admission: Scale Hostility	Score in the PANSS item ‘hostility’ at the time of admission to the referenced hospital
PA8	PANSS at admission: Scale Blunted affect	Score in the PANSS item ‘blunted affect’ at the time of admission to the referenced hospital
PA9	PANSS at admission: Scale Emotional withdrawal	Score in the PANSS item ‘emotional withdrawal’ at the time of admission to the referenced hospital
PA10	PANSS at admission: Scale Poor rapport	Score in the PANSS item ‘poor rapport’ at the time of admission to the referenced hospital
PA11	PANSS at admission: Scale Passive/apathetic social withdrawal	Score in the PANSS item ‘passive/apathetic social withdrawal’ at the time of admission to the referenced hospital
PA12	PANSS at admission: Scale Difficulty in abstract thinking	Score in the PANSS item ‘difficulty in abstract thinking’ at the time of admission to the referenced hospital
PA13	PANSS at admission: Scale Lack of spontaneity and flow of conversation	Score in the PANSS item ‘lack of spontaneity and flow of conversation’ at the time of admission to the referenced hospital
PA14	PANSS at admission: Scale Stereotyped thinking	Score in the PANSS item ‘stereotyped thinking’ at the time of admission to the referenced hospital
PA15	PANSS at admission: Scale Somatic concern	Score in the PANSS item ‘somatic concern’ at the time of admission to the referenced hospital
PA16	PANSS at admission: Scale Anxiety	Score in the PANSS item ‘anxiety’ at the time of admission to the referenced hospital
PA17	PANSS at admission: Scale Guilt feelings	Score in the PANSS item ‘guilt feelings’ at the time of admission to the referenced hospital
PA18	PANSS at admission: Scale Tension	Score in the PANSS item ‘tension’ at the time of admission to the referenced hospital
PA19	PANSS at admission: Scale Mannerisms and posturing	Score in the PANSS item ‘mannerism and posturing’ at the time of admission to the referenced hospital

PA20	PANSS at admission: Scale Depression	Score in the PANSS item ‘depression’ at the time of admission to the referenced hospital
PA21	PANSS at admission: Scale Motor retardation	Score in the PANSS item ‘motor retardation’ at the time of admission to the referenced hospital
PA22	PANSS at admission: Scale Uncooperativeness	Score in the PANSS item ‘uncooperativeness’ at the time of admission to the referenced hospital
PA23	PANSS at admission: Scale Unusual thought content	Score in the PANSS item ‘unusual thought content’ at the time of admission to the referenced hospital
PA24	PANSS at admission: Scale Disorientation	Score in the PANSS item ‘disorientation’ at the time of admission to the referenced hospital
PA25	PANSS at admission: Scale Poor attention	Score in the PANSS item ‘poor attention’ at the time of admission to the referenced hospital
PA26	PANSS at admission: Scale Lack of judgement and insight	Score in the PANSS item ‘lack of judgement and insight’ at the time of admission to the referenced hospital
PA27	PANSS at admission: Scale Disturbance of volition	Score in the PANSS item ‘disturbance of volition’ at the time of admission to the referenced hospital
PA28	PANSS at admission: Scale Poor impulse control	Score in the PANSS item ‘poor impulse control’ at the time of admission to the referenced hospital
PA29	PANSS at admission: Scale Preoccupation	Score in the PANSS item ‘preoccupation’ at the time of admission to the referenced hospital
PA30	PANSS at admission: Scale Active social avoidance	Score in the PANSS item ‘active social avoidance’ at the time of admission to the referenced hospital
PA_A	PANNS Score at admission	PANSS total score at the time of admission to the referenced hospital
PA32	PANSS at discharge: Scale Delusion	Score in the PANSS item ‘delusion’ at the time of discharge from the referenced hospital
PA33	PANSS at discharge: Scale Conceptual disorganization	Score in the PANSS item ‘conceptual disorganisation’ at the time of discharge from the referenced hospital
PA34	PANSS at discharge: Scale Hallucinations	Score in the PANSS item ‘hallucinations’ at the time of discharge from the referenced hospital
PA35	PANSS at discharge: Scale Hyperactivity	Score in the PANSS item ‘hyperactivity’ at the time of discharge from the referenced hospital
PA36	PANSS at discharge: Scale Grandiosity	Score in the PANSS item ‘grandiosity’ at the time of discharge from the referenced hospital

PA37	PANSS at discharge: Scale Suspiciousness/persecution	Score in the PANSS item 'suspiciousness/persecution' at the time of discharge from the referenced hospital
PA38	PANSS at discharge: Scale Hostility	Score in the PANSS item 'hostility' at the time of discharge from the referenced hospital
PA39	PANSS at discharge: Scale Blunted affect	Score in the PANSS item 'blunted affect' at the time of discharge from the referenced hospital
PA40	PANSS at discharge: Scale Emotional withdrawal	Score in the PANSS item 'emotional withdrawal' at the time of discharge from the referenced hospital
PA41	PANSS at discharge: Scale Poor rapport	Score in the PANSS item 'poor rapport' at the time of discharge from the referenced hospital
PA42	PANSS at discharge: Passive/apathetic social withdrawal	Score in the PANSS item 'passive/apathetic social withdrawal' at the time of discharge from the referenced hospital
PA43	PANSS at discharge: Scale Difficulty in abstract thinking	Score in the PANSS item 'difficulty in abstract thinking' at the time of discharge from the referenced hospital
PA44	PANSS at discharge: Scale Lack of spontaneity and flow of conversation	Score in the PANSS item 'spontaneity and flow of conversation' at the time of discharge from the referenced hospital
PA45	PANSS at discharge: Scale Stereotyped thinking	Score in the PANSS item 'stereotyped thinking' at the time of discharge from the referenced hospital
PA46	PANSS at discharge: Scale Somatic concern	Score in the PANSS item 'somatic concern' at the time of discharge from the referenced hospital
PA47	PANSS at discharge: Scale Anxiety	Score in the PANSS item 'anxiety' at the time of discharge from the referenced hospital
PA48	PANSS at discharge: Scale Guilt feelings	Score in the PANSS item 'guilt feelings' at the time of discharge from the referenced hospital
PA49	PANSS at discharge: Scale Tension	Score in the PANSS item 'tension' at the time of discharge from the referenced hospital
PA50	PANSS at discharge: Scale Mannerisms and posturing	Score in the PANSS item 'mannerisms and posturing' at the time of discharge from the referenced hospital
PA51	PANSS at discharge: Scale Depression	Score in the PANSS item 'depression' at the time of discharge from the referenced hospital
PA52	PANSS at discharge: Scale Motor retardation	Score in the PANSS item 'motor retardation' at the time of discharge from the referenced hospital
PA53	PANSS at discharge: Scale Uncooperativeness	Score in the PANSS item 'uncooperativeness' at the time of discharge from the referenced hospital

PA54	PANSS at discharge: Scale Unusual thought content	Score in the PANSS item ‘unusual thought content’ at the time of discharge from the referenced hospital
PA55	PANSS at discharge: Scale Disorientation	Score in the PANSS item ‘disorientation’ at the time of discharge from the referenced hospital
PA56	PANSS at discharge: Scale Poor attention	Score in the PANSS item ‘poor attention’ at the time of discharge from the referenced hospital
PA57	PANSS at discharge: Scale Lack of judgement and insight	Score in the PANSS item ‘lack of judgement and insight’ at the time of discharge from the referenced hospital
PA58	PANSS at discharge: Scale Disturbance of volition	Score in the PANSS item ‘disturbance of volition’ at the time of discharge from the referenced hospital
PA59	PANSS at discharge: Scale Poor impulse control	Score in the PANSS item ‘poor impulse control’ at the time of discharge from the referenced hospital
PA60	PANSS at discharge: Scale Preoccupation	Score in the PANSS item ‘Preoccupation’ at the time of discharge from the referenced hospital
PA61	PANSS at discharge: Scale Active social avoidance	Score in the PANSS item ‘active social avoidance’ at the time of discharge from the referenced hospital
PAS_D	PANNS Score at discharge	PANSS total score at the time of discharge from the referenced hospital
